# Composite Coatings of Chitosan and Alginate Emulsions with Olive Oil to Enhance Postharvest Quality and Shelf Life of Fresh Figs (*Ficus carica* L. cv. ‘Pingo De Mel’)

**DOI:** 10.3390/foods10040718

**Published:** 2021-03-29

**Authors:** Tiago M. Vieira, Margarida Moldão-Martins, Vítor D. Alves

**Affiliations:** Linking Landscape, Environment, Agriculture and Food (LEAF) Research Centre, Instituto Superior de Agronomia (ISA), Universidade de Lisboa, Tapada da Ajuda, 1349-017 Lisboa, Portugal; tdmvieira@gmail.com (T.M.V.); vitoralves@isa.ulisboa.pt (V.D.A.)

**Keywords:** alginate, chitosan, edible coatings, crosslinking, postharvest, figs, shelf life

## Abstract

Fresh figs are very appreciated and have been associated with health benefits. However, these fruits are highly perishable. In this study, edible coatings were studied envisaging their positive effect in enhancing figs’ shelf-life. Fig fruits cv. ‘Pingo de mel’ were harvested at commercial ripening stage and single emulsion-based coatings, composed of chitosan + olive oil and alginate + olive oil, were applied. After coatings application by dipping each fruit in the emulsion-based solutions at 4 °C and drying, the coated fruits were sprayed with crosslinking solutions (6% tripolyphosphate and 1% calcium chloride for chitosan and alginate-based coatings, respectively). Then, were maintained at 4 °C and analyzed after 1, 7, 14 and 19 days of storage. After each time interval, fruits were further maintained at 25 °C for 2 days. The results have shown that coatings were effective on delaying fungal decay and postharvest ripening indicators (respiration rate, mass loss, softening and total soluble solids/titratable acidity ratio). The results foresee a fruits’ shelf life between 14 and 19 days under refrigeration at 4 °C that may be followed up to 2 days at ambient temperature, higher than that estimated for uncoated fruits (less than 14 days at 4 °C plus to 2 days at ambient temperature).

## 1. Introduction

The fig (*Ficus carica* L.) is native to western Asia and has been an important crop worldwide for dry and fresh consumption [1]. The production of this fruit is mainly located in Turkey, Egypt, Algeria, and Morocco, which accounts 65% of the world production, but it is also well spread through the Mediterranean basin, USA (California), Brazil, India and Japan [2,3,4,5].

The fig is one of the most abundant fruits in the Mediterranean diet, containing considerable amounts of amino acids, polyphenols, several carotenoids, vitamins, dietary fibers, polyunsaturated fats and minerals (e.g., such as potassium, calcium and iron). Figs are free of sodium, and like other fruits, cholesterol-free [1,2,3,6]. The Mediterranean diet achieved by consuming fresh salads, vegetables, fruits, and their derived products, has been reported to promote health and quality of life in those who adhere to it, specifically by preventing pathophysiological conditions related to coronary heart disease, cancer and diabetes. Along with olive, the fig is a characteristic and abundant fruit in this diet [6].

The fig is considered a climacteric fruit [3,7] and so depending on the maturity stage at which the fruit is harvested [2], it exhibits autocatalytic synthesis of ethylene and a respiratory upsurge which affects its commercial quality, promoting senescence with typical effects of an increase in the rate of yellowing, increase in microbial growth, induction of physiological disorders, particularly chilling effects, and development of undesirable flavors [8]. Recently, an increase of consumer demand for fresh quality produce of less familiar fruits, consequently increased the fresh fig production. However, the fig is very sensitive to microbial growth of bacteria, molds and yeasts even when stored at low temperature [9,10].

The use of a modified atmosphere (MAP) has been reported to reduce microbial proliferation, along with respiration rate and metabolic activity, with the benefit of delaying senescence [10]. In this sense, edible coatings can provide an alternative method to design the passive modified atmosphere created in the package over time, due to their barrier properties, reducing the quality changes and quantity losses during storage, which may contribute to extend the product shelf life [11]. Edible coatings have also a high potential to carry active ingredients such as anti-browning agents, colorants, flavors, nutrients and antimicrobial compounds that can extend product shelf life and reduce risk of pathogen growth on the food surface [11]. Moreover, edible coatings or edible/biodegradable films are important solutions for the reduction of synthetic packaging waste, because of their biodegradable raw materials [12,13,14].

Edible coatings are thin layers of edible materials applied on the products surface in addition to or as a replacement for natural protective waxy coatings to provide a barrier to moisture, solutes or gases, water/lipid solubility, and other functional characteristics, for example, color, enhanced appearance and peel mechanical properties [3]. These coatings are usually made of proteins, polysaccharides and lipids (e.g., waxes). While the former materials are able to produce coatings and films with high barrier properties to gases (e.g., oxygen and carbon dioxide) and good mechanical properties when exposed to low relative humidity conditions, this good performance is highly negatively affected in environments with high relative humidity and moisture content due to their hydrophilic character. Lipid-based coatings and films, made from a wide array of substances, such as animal and plant waxes, vegetable oils and fatty acids are hydrophobic and resistant to moisture [13]. However, some waxes tend to produce thicker and more brittle coatings and films [13]. As such, composite bilayer or emulsion-based coatings and films, combining the properties of hydrophilic biopolymers such as chitosan and sodium alginate and the hydrophobic character of lipids, have been developed [3,13,15].

Alginate has unique colloidal properties and its ability to form strong gels or an insoluble structure upon reaction with multivalent metal cations, like calcium, makes it suitable for use as a fruit coating material [15]. The effectiveness of alginate coatings to improve the quality and shelf life of different fruits, such as strawberries and fresh-cut cantaloupe melon [16,17], has been evaluated. In the other hand, chitosan is a cationic polysaccharide, derived from the partial deacetylation of chitin (the main constituent of crustaceans’ exoskeleton), and possess several important advantages including, intrinsic antimicrobial activity, strength and resistance upon handling that may be enhanced by crosslinking reactions, besides being non-toxic and biodegradable like other natural polysaccharides [18]. Chitosan coatings have been used on fruit and vegetable products such as strawberries, apples and cucumbers, for antimicrobial protection, as well as in films to prolong the shelf life of banana, mango and capsicum [3,19].

In emulsified systems, lipids are dispersed and trapped in the biobased polymer matrix [13]. Many researchers have developed composite emulsion-based films and coatings in recent years. These studies involved the use of sodium alginate, gelatin and canola oil [20], monolayer chitosan and carrageenan with sunflower oil to coat longan fruits [21], sodium alginate with sunflower oil to coat cantaloupe and strawberries [17], chitosan based film with clove and melaleuca essential oils [22], sodium alginate with soybean oil to coat fresh cucumber [23], gelatin with frog skin oil to coat persimmon fruits [24] and composite chitosan with carnauba wax and oregano essential oil [25]. In addition, coatings based on chitosan, sodium alginate, and olive oil, applied to coat fresh whole figs have shown to be a useful postharvest technology in preserving not only the organoleptic and sensory attributes but also bioactive components of these fruits during storage at low temperature [3,26].

In a recent study [27] were developed and characterized two monolayer emulsion based edible films with chitosan and alginate, with olive oil (25% and 100% of biopolymer basis, respectively), using soybean lecithin as surfactant (25% of lipid basis). After drying, the films were sprayed with a crosslinking solution (6% *w/v* tripolyphosphate (TPP) and 6% *w/v* CaCl_2_, respectively). Olive oil was chosen due to its interesting properties. It is a vegetable oil with a very high monounsaturated fatty acid content (56.3–86.5%, particularly oleic acid), and is rich in tocopherols and phenolic substances which act as antioxidants, being associated with various positive health benefits. Besides, it is easily available, non-depletable and non-volatile [13,28]. Therefore, it is an obvious alternative to consider when dealing with edible films aimed at reducing water vapor transmission. Overall, the developed films presented a good potential as barriers to water vapor and increased resistance to liquid water, which envisages the use of such formulations to produce either biodegradable/edible films or edible coatings.

In this sense, the current work aimed to test for the first time the application of monolayer edible chitosan and alginate -based emulsions with olive oil, as coatings on whole fresh figs (*F. carica*, var. ‘Pingo de mel’ quite appreciated in Portugal), and study their potential for extending fruits shelf life, by assessing the influence of these types of coatings on fruit physiology (respiratory profile), fungal decay and quality attributes (weight loss, firmness, color, titratable acidity and soluble solids) over time. The attention was driven to evaluate the post-harvest quality of coated figs over time, not only under the common refrigerated conditions (4 °C), but also during two days at room temperature after the refrigeration storage period, in order to evaluate the consequences of a less appropriate handling temperature after purchase. 

## 2. Materials and Methods

### 2.1. Plant Material and Chemicals

Figs cv. ‘Pingo de mel’ were harvest (handpicked) early in the morning, at commercial ripening stage, in September 2018, from an orchard of Santarém district, Portugal (39.507381, −8.642617). Fruits were maintained under ambient conditions (22–25 °C) for about 8 h before the essays, due to the logistic conditions available. 

For coating solutions preparation, chitosan and sodium alginate were purchased from Golden/Shell Biochemical Co. Ltd. (Zhejiang, China) and Quimidroga, S.A. (Barcelona, Spain), respectively. Glycerol was acquired from Fisher Scientific (Loughborough, UK), lactic acid from Panreac Quimica SAU (Barcelona, Spain), and calcium chloride from Absolve^®^ (Portugal). The soybean lecithin was purchased from Tokyo Chemical Industry Co., Ltd. (Tokyo, Japan), and olive oil (Lisbon, Portugal) was acquired at a local store.

### 2.2. Coating Emulsions Preparation and Viscosity Evaluation

Chitosan and alginate-based emulsions with olive oil were produced according to the method described by Vieira, Moldão-Martins and Alves [27], and applied as coatings on the selected fruits. Briefly, coating solutions composed of 2% *w/v* chitosan (C) (dissolved in lactic acid 1% *v*/*v*) and 1% *w/v* sodium alginate (A) (dissolved in deionized water) were prepared, to which 25% olive oil (OO) (*w*/*w* biopolymer basis) and 25% soybean lecithin (surfactant, *w*/*w* lipid basis) were added for chitosan emulsions (C-OO), and 100% *w*/*w* olive oil and 25% *w*/*w* surfactant for alginate emulsions (A-OO). Glycerol was used as plasticizer (25% for chitosan and 50% for alginate emulsions, *w*/*w* biopolymer basis). The emulsions were obtained by stirring the mixtures with an Ultraturrax T25 basic (IKA^®^ Works, Inc., Wilminton, NC, USA) at 13,500 rpm for 2 min.

Tests were carried out to study the viscosity properties of the prepared coating emulsions using a controlled stress rheometer (Haake Mars III—Thermo Scientific, Karlsruhe, Germany), with a UTC-Peltier system to control temperature, and a cone-plate sensor system (angle 2°, diameter 35 mm). Measurements were carried out using a stationary shear flow, according to an adapted method from [29]. The apparent viscosity was recorded as a function of shear rate (0.01–1000 s^−1^). It was measured at 4 °C (refrigeration temperature) an at 25 °C (room temperature). 

The Cross model was simplified (Equation (1)), in which the infinite shear viscosity (Ƞ∞) was considered negligible, assuming Ƞa >> Ƞ∞ and Ƞ0 >> Ƞ∞, and used for modelling the obtained apparent viscosity–shear rate curves:

(1) $$Ƞa=\frac{Ƞ0}{1+{\left(\lambda \cdot \dot{\gamma }\right)}^{m}}$$

where Ƞ*a* is the apparent viscosity (Pa·s), Ƞ0 is the viscosity on the first Newtonian plateau (Pa·s), λ is a time constant (s), $\dot{\gamma }$ is the shear rate (s^−1^), m is correlated to the flow index (n) by m = 1 − n and it takes a value of 0 for Newtonian behavior, and values above 0 for shear thinning fluids. Besides, the coefficient of determination (R^2^), the standard deviation of the estimate (SDE) with the mean relative error (MRE), were considered to evaluate the goodness of fit adjustment of the model selected.

### 2.3. Sample Preparation and Storage Conditions

Selected fig fruits (17 kg), homogeneous in color and size, and without visual defects, were randomly distributed into three groups of 184 fruits each. The first group was the control group, without application of coating emulsion (Ctr), the second group was coated with chitosan/olive oil emulsion (C-OO) and the third group was coated with sodium alginate/olive oil emulsion (A-OO). The coatings were applied by dipping each fruit in the coating solutions at 4 °C for 30 s. In order to avoid handling injuries, the fruits were held by the stem during dipping. After the dipping process, the fruits were dried using ventilated air for 15 min at room temperature. 

Following that, each coated fruit from the second and third groups was sprayed with a crosslinking solution: calcium chloride 6% *w*/*v* for sodium alginate/olive oil coating (A-OO-Ca), in order to add 3.2 mg Ca^2+^/cm^2^ dry surface, and tripolyphosphate 6% *w*/*v* for chitosan/olive oil coating (C-OO-TPP), in order to add 4 mg TPP/cm^2^ dry surface, to enhance coatings integrity by reducing absorption of condensed water vapor that may occur over the fruit surface, given the storage conditions applied [27]. The fruits with crosslinked coatings were dried for 15 min at room temperature. 

All figs, coated and uncoated, were stored at 4 °C and 70% RH. Fig samples of each group were analyzed at a defined time of the storage period (days 0, 1, 7, 14, and 19) right after being removed from refrigerated conditions, and again after 2 days at ambient temperature (around 25 °C). Fruit analysis at the defined days involved visual infection, respiratory behavior and physical-chemical properties (weight loss, firmness, color, soluble solids and titratable acidity) that were measured as described below. 

### 2.4. Analytical Control of Fruits

#### 2.4.1. Fruit Fungal Infection and Disease Incidence

The fruit fungal infection was evaluated as described by [3]. Fruits from all groups were visually inspected to detect visual mold growth during storage, reporting as infected figs those fruits with mycelia development, brown spots, and softening of the infected area. A number of 12 figs per group was used. The results from fungal incidence were expressed as the percentage of infected fruits of the hole sample (12 figs).

#### 2.4.2. Respiration Rate

Five fruits of each group were placed in a 1.01 L closed flask for 2 h and the changes in the headspace gas composition were measured using a gas analyzer (Checkmate 9900, PBI Dansensor, Ringsted, Denmark). Fruit respiration rate (RT; mmol CO_2_ kg^−1^ h^−1^) was calculated considering the flask void volume (V; mL), fruit mass (m; kg), time of closure (t; h), and variation in CO_2_ concentration inside the container (% ΔCO_2_) (Equation (2)). Five replicates were performed per group and storage time.

(2) $$RT=\frac{\Delta CO2}{100}\;\times \;V\;\times \;\frac{1000}{m}\;\times \;\frac{60}{t}$$

#### 2.4.3. Weight Loss

Fig samples of each group were weighed at the beginning of the experiment, after the indicated storage periods at 4 °C, and after two days at 25 °C. Cumulative weight loss was expressed as the percentage loss from the initial weight. Five replicates (of two figs each) were measured per group.

#### 2.4.4. Firmness and Surface Color

A Texturometer (TA-XT2, Stable Micro System, Surrey, UK) with a 5 kg load cell, equipped with a cylindrical 2 mm diameter probe, was used to evaluate the firmness in whole figs (adapted from [3] method). Firmness (maximum force upon puncture) was measured on five replicates (of two figs each) per group at each storage time. The equatorial zone of each fruit was penetrated (10 mm depth) with a constant speed of 5 mm min^−1^. All fruit samples were conditioned at 25 °C before measurements. 

The color alterations on the skin of the fruits caused by the application of coatings and storage time were evaluated by measuring the surface color six times (on the upper, middle and bottom surface) of five replicates (of two figs each) from each group. A colorimeter (Konica Minolta CR-300, Williams Drive Ramsey, NJ, USA) was used, and the CIE L*a*b* color space was applied. Total color differences (ΔE) for a given sampling time was calculated in relation to the color of the raw material on day 0, according to Equation (3).

(3) ΔE=[(L0*− L*)2+(a0*− a*)2+(b0*− b*)2]12 

where the subscript 0 refers to parameter value at day 0.

#### 2.4.5. Total Soluble Solids and Titratable Acidity 

Four fruits of each group were homogenized using an Ultra-Turrax blender (IKA T18 basic Ultra-Turrax, Staufen, Germany) at 12,000 rpm for 10 s and the homogenate was used to measure the total soluble solids (TSS) and titratable acidity (TA), reporting the mean value of three replicates.

The TSS (expressed as ˚Brix) value was determined using a refractometer (Atago, Fisher Scientific, Ga., Bellevue, WA, USA), with 1 mL of juice obtained squeezing the homogenate through a double layer of gauze. 

TA was evaluated following the methodology of the [30]. Twenty grams of the homogenized sample were mixed with 100 mL of distilled water and the mixture was filtered. TA was determined by titration of the permeate with 0.1 N NaOH (to a pH value of 8.1 ± 0.2) and was expressed as the percentage of citric acid (TA, % of citric acid = g citric acid/100 g fresh weight (FW)).

### 2.5. Statistical Analysis and Adjusts

Statistica 8.0 software (Statsoft Inc., Tulsa, OK, USA) was used for statistical analysis. Analysis of variance (ANOVA) and Tukey’s multiple range test (*p* level of 0.05) to detect differences among mean values of films properties was used. Model adjustments were made using OriginPro 8.0 software (Origin Lab, Northampton, MA, USA).

## 3. Results and Discussion

### 3.1. Viscosity Properties of the Coating Emulsions

Rheological analysis of coating solutions is useful technologically to identify the most appropriate coating system as well as to optimize operating conditions. In fact, the thickness of the applied coating is highly dependent on the coating solution viscosity. Generally, the viscosity of an emulsion is strongly dependent on the concentration of the dispersed phase [31], and for such systems, the relative amounts of polymer and oil phases become a key development issue in influencing the rheological properties of an emulsion [32].

#### 3.1.1. Apparent Viscosity Curves

**Figure 1:**
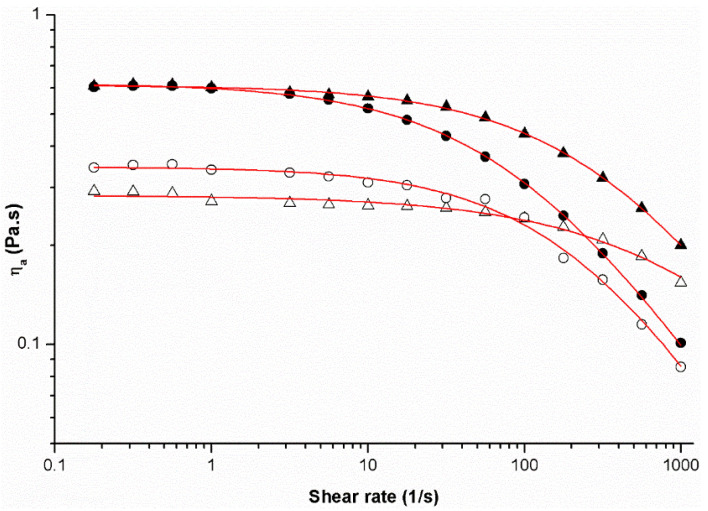
Viscosity flow curves of the emulsions: chitosan (C-OO) (triangular plots) and alginate (A-OO) (circular plots), at temperatures of 4 °C (solid plots) and 25 °C (open plots). Continuous lines represent the predictions of the simplified Cross model.

Figure 1 shows the apparent viscosity as a function of shear rate of all coating emulsions prepared. The steady-shear behavior of the chitosan (C-OO) and alginate (A-OO) emulsions indicated a typical viscosity behavior of many fluids (e.g., polymeric solutions, flocculated dispersions, colloids) [31], showing a Newtonian plateau followed by a shear thinning behavior with increasing shear rate.

The behavior of these samples can be explained by changes in the molecule organization (e.g., entanglements and hydrogen, electrostatic and hydrophobic bonds), when energy was applied, which influenced the formation or breaking of such interactions [33]. Thus, at lower shear rates the breaking of entanglements by the forced shear was equal to the formation of new ones and no changes were reflected, the Newtonian plateau occurring when the viscosity presented a constant value (η0). For high shear rates, the rate of disruption of entanglements predominates in relation to the rate of formation of new ones, a partial alignment of the molecules that leads to a decrease of the apparent viscosity occurring with the increase of shear rate. As result, there was the transition from Newtonian to shear-thinning behavior, resulting in a shear-dependent behavior [34,35]. 

The high determination coefficient (>0.971), low value of mean relative error (3.2–6.1%), and low standard deviation of the estimate (0.024–0.032) confirmed good concordance of the modified Cross model (Equation (1)) with the experimental data of Figure 1. The estimated values for the parameters Ƞ0, λ and m of the emulsions are summarized in Table 1.

#### 3.1.2. Effect of Temperature on Apparent Viscosity

The effect of temperature (4° and 25 °C) on the steady shear behavior of the studied emulsions was evaluated. The apparent viscosity increased with decreasing emulsions temperature. The Newtonian plateau at lower shear rates approached a similar value for C-OO and A-OO emulsions at 4 °C (0.612 and 0.620 Pa·s, respectively), which was higher to that obtained at 25 °C (0.283 and 0.346 Pa·s, respectively) (Table 1). This increase of viscosity upon decreasing temperature was expected for fluids, including emulsions. The time constant (λ) is a relaxation time and was related to the transition from the Newtonian plateau to the shear thinning region. As commonly observed for polymer systems, for the emulsions analyzed this transition occurred at higher shear rates (and lower relaxation time) as the viscosity increased [33]. 

The emulsions of this work were composed of different biopolymers at different concentrations, containing also different contents of olive oil and surfactant, surely presenting a diverse microstructure, namely in terms of oil droplets size and consistency of the continuous phase [36]. Though, the overall contribution of such factors resulted in a similar viscosity behavior, at both temperatures, regarding the values of the Newtonian plateau and relaxation time. Higher differences were only observed in the shear-tinning region. 

The results suggested that both emulsions possessed viscosity properties that enabled their application as coatings of figs by immersion. However, the application of such emulsions at a lower temperature (4 °C) may have favored the adhesion of a thicker coating on the surface of the fruits. In addition, the maintenance of the fruits under refrigerated conditions as much time as possible after harvesting was of major importance for their preservation. In this sense, the emulsions were applied as coatings in the selected fig fruits at a temperature of 4 °C.

### 3.2. Effect of Coatings Application on Figs Quality under Storage

#### 3.2.1. Fruit Fungal Infections and Disease Incidence

**Figure 2:**
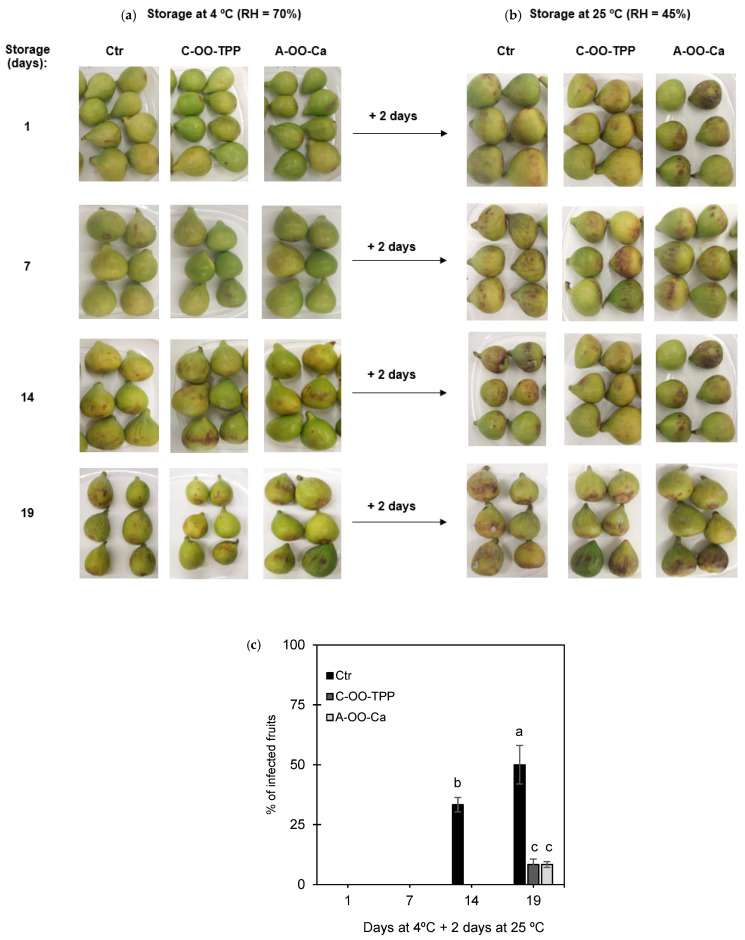
Figs (*Ficus carica* L. var. ´Pingo de mel´) with crosslinked chitosan-olive oil and alginate-olive oil emulsion-based coatings (C-OO-TPP and A-OO-Ca, respectively) and uncoated control (Ctr), during storage at 4 °C (**a**), and after being transferred to room temperature for 2 days (**b**); fungal disease incidence (**c**). Means followed by equal letters, after storage, do not differ by Tukey’s test at 5% of error probability. RH: Relative humidity.

Figure 2 shows the visual appearance of uncoated (Ctr) and coated fig fruits (C-OO-TPP and A-OO-Ca) maintained under storage at 4 °C (Figure 2a) and after 2 days at room temperature (Figure 2b), as well as the fungal disease infection (% of infected fruits) (Figure 2c). 

The application of refrigerated conditions (4 °C) on fresh figs (coated and uncoated) during the storage period slowed down the incidence of visual degradation (Figure 2a). It should be noted that the coatings were not perceptible, and the fruits kept in the refrigerated chamber (4 °C) did not show any visual differences between groups in the first 14 days of storage. The higher difference was observed in the stability at room temperature after being removed from the refrigerated chamber. The coated fruits had good stability, without visual infections, at room temperature for two days after 14 days of storage under refrigeration. On the contrary, the uncoated fruits only presented this stability until the 7th day of cold storage. For these fruits, a high percentage of affected fruits (33.3%) was observed after 14 days of cold storage plus 2 days at ambient temperature, increasing significantly at the end of 19 days of cold storage plus 2 days at ambient temperature (Figure 2b,c). The application of the coatings retarded the fruits visual contamination (Figure 2b,c), and a visual decay in coated fruits was only detected after 19 days of storage plus 2 days at ambient temperature. At these instant, coated figs presented a visual fungal contamination on approximately 5% of the fruits, whereas about 50% of the uncoated figs were contaminated. Reyes-Avalos et al. [3] reported that edible alginate-chitosan bilayer coatings were capable of reducing the fungal contamination (<5%) of fresh figs during the 15 days of storage at 6 °C. In the present work, the results were even more promising, as a quite low fungal infection was also achieved using single layer coatings and after a longer storage period, including 2 days at room temperature. Other results on microbial growth delay in fruits were also reported. As examples, Romanazzi, et al. [37] have shown that chitosan coatings were capable of reducing microbial contamination in strawberries stored at 20 °C and 95–96% RH for 4 days. Additionally, Moayednia, et al. [38] demonstrated that alginate coatings and their combination with low temperature (5 °C) reduced visual decay in strawberry fruit during 14 days of storage. In addition, coatings based on chitosan and propolis were tested in the control of infection by *Aspergillus flavus* in fig fruits [39]. The results showed that the coatings reduced the development of this fungus, as well as aflatoxin production, under controlled laboratory and semi-commercial conditions.

#### 3.2.2. Respiration Rate

The respiration rate was evaluated through the CO_2_ production and provides essential information about fruit metabolic activity and can be used to predict the shelf life of fruits and vegetable products [40]. 

**Figure 3:**
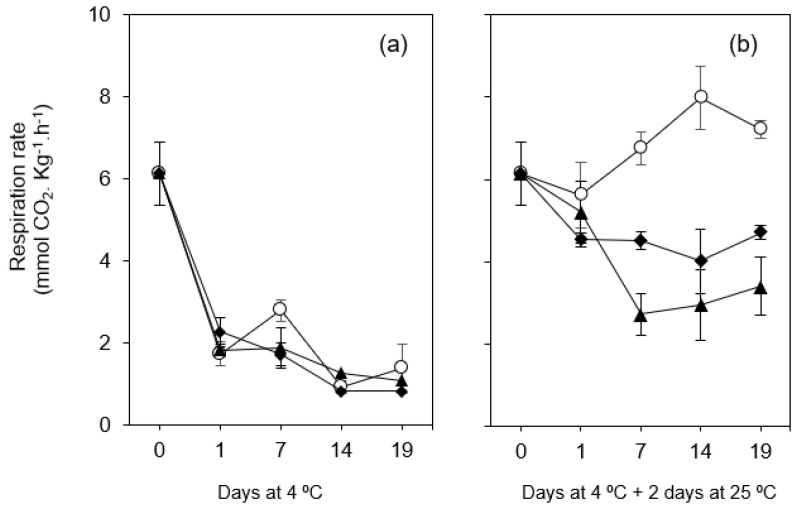
Respiration rate of fresh figs (*Ficus carica* L. var. ´Pingo de mel´) coated (◆ C-OO-TPP and ⯅ A-OO-Ca) and uncoated (○ Ctr), under refrigeration over 19 days at 4 °C (**a**) and after being transferred to room temperature for 2 days (**b**). Each symbol represents each experimental condition, which is associated to a line.

In this study, figs under cold storage (4 °C) regardless of the treatment applied (with or without coating), show a significant reduction of CO_2_ emission (by up 67%) in the first day of storage, reaching values lower than 2.0 mmol CO_2_ kg^−1^ h^−1^ during most of the remaining storage period (Figure 3a). On the other hand, when fruits were maintained at ambient temperature for 2 days after the storage period at 4 °C, all fruits showed higher respiration rate values (Figure 3b). However, a different behavior was observed between coated and uncoated fruit groups. While for the former the CO_2_ release measured after 2 days at 25 °C decreased with increasing the previous storage period at 4 °C, for the later a substantial increase of respiration rate was observed. After 19 days at 4 °C plus 2 days at 25 °C, the respiration rate was 4.70 and 3.40 mmol CO_2_ kg^−1^ h^−1^ for coated fruits (C-OO-TPP and A-OO-Ca, respectively) and 7.21 mmol CO_2_ kg^−1^ h^−1^ for uncoated ones, for which a peak was observed after 14 days at 4 °C plus 2 days at 25 °C (7.98 mmol CO_2_ kg^−1^ h^−1^). 

Climacteric fruits during ripening showed a sudden increase in respiration rate followed by a decline, which was the major cause of their short shelf life. The lower respiration rate in C-OO-TPP and A-OO-Ca coated fruits may have been due to reduced gas interchange between the fruits and the environment, and subsequently, low availability of oxygen for respiration. The suppressed climacteric respiration peak observed in both coated fruits could be due to insufficient carbon dioxide permeation and its accumulation in the fruits, which inhibited ethylene synthesis and action such as the ripening process was delayed [41]. Several authors have also reported a reduction in the CO_2_ production in coated fruits, including pears [42], longan fruit [43], nectarine [44], sweet cherry fruit [45], mangoes [40], as well fresh figs [3].

#### 3.2.3. Weight Loss

**Figure 4:**
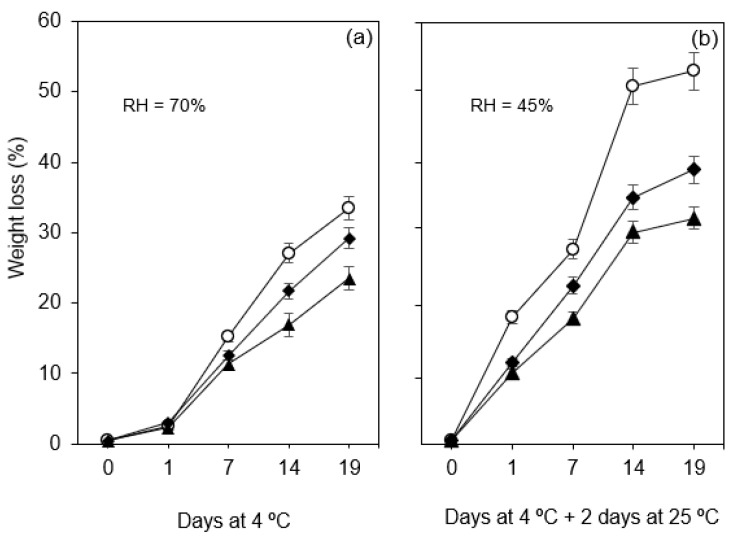
Weight loss of fresh figs (*Ficus carica* L. var. ´Pingo de mel´) coated (◆ C-OO-TPP and ⯅ A-OO-Ca) and uncoated (○ Ctr), under refrigeration for over 19 days at 4 °C (**a**) and after being transferred to room temperature for 2 days (**b**). RH: Relative humidity.

The weight loss of fruit and vegetables is closely related to transpiration and respiration processes, being considered one of the most critical factors in the loss of quality [46]. Figure 4 shows the values of weight loss of uncoated and coated fruit samples stored under refrigeration at 4 °C over 19 days, and after 2 days at room temperature (25 °C).

As can be seen, the weight loss was high during the storage period, and even more pronounced when the samples were kept at room temperature for 2 days after leaving the refrigerated chamber. Though, the results show that both coatings were able to reduce the weight loss over time when comparing to the values obtained for uncoated fruits. The maximum value was observed for uncoated figs after 19 days at 4 °C of storage (33.4% of initial weight, Figure 4a). For the same time period, values of 29.2% and 23.5% were observed for fruit with C-OO-TPP and A-OO-Ca coatings, respectively. 

When uncoated fruit samples were transferred to room temperature (25 °C) and relative humidity conditions (45%) after the refrigeration storage period (Figure 4b), a maximum of weight loss of 55% was registered in the end of the storage period. As already mentioned, the application of coatings was also crucial after transferring the samples to room temperature and relative humidity conditions. In this case, both coatings had a significant effect on reducing the weight loss of figs, by 15.69% for crosslinked chitosan-based emulsion (C-OO-TPP) and 22.66% for crosslinked alginate-based emulsion (A-OO-Ca), after 2 days at ambient conditions (Figure 4b).

Weight loss of fruits is due to the gradient of water vapor pressure between the fruit and the surrounding air, which is usually reduced by both epidermal cell layer and cuticle [45]. However, the edible coating acts as an extra layer which also coats the stomata leading to a decrease in transpiration and consequently to a reduction in weight loss, this being the primary beneficial effect of edible coatings [45]. In fact, enhanced barrier properties of the emulsion-based coatings used in this work were envisaged to be present according to the water vapor permeability results obtained previously for films produced with the same emulsions [27]. The higher water vapor barrier properties of emulsion-based films have been reported by other authors [3,20,21,22,23,24,28,35,47]. 

#### 3.2.4. Firmness and Surface Color

**Figure 5:**
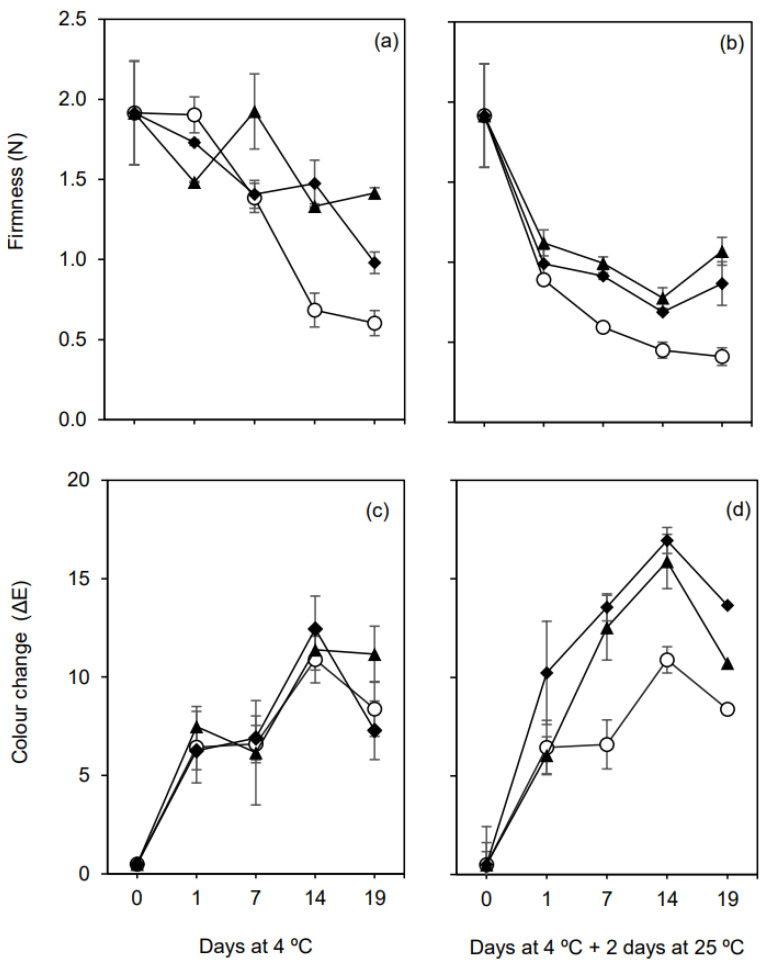
Firmness (N) and colour changes on the skin of the fruits (ΔE) for figs (*Ficus carica* L. var. ´Pingo de mel´) coated (◆ C-OO-TPP and ⯅ A-OO-Ca) and uncoated (○ Ctr), under refrigeration for over 19 days at 4 °C (**a**,**c**) and after being transferred to room temperature for 2 days (**b**,**d**). Each symbol represents each experimental condition, which is associated to a line.

Firmness and skin color change (ΔE) of fruits during storage at refrigerated conditions (4 °C), and after leaving the refrigerated chamber and maintained at room temperature (25 °C) for 2 days, are shown in Figure 5.

In general, there was a significant decrease in the firmness values of all samples throughout the storage time (Figure 5a,b). However, the results showed that both coatings were able to reduce figs softening over time when comparing to uncoated fruits. This fact was more evident after the 7th day, for fruits during storage under refrigeration (Figure 5a), and for fruit samples analyzed 2 days at room temperature after a period under refrigeration (Figure 5b). The crosslinked chitosan-based and alginate-based emulsion coatings did significantly (*p* < 0.05) delay the loss of firmness after 19 days of cold storage or after transferring the fruits for ambient conditions (25 °C). In the latter case, after 19 days at 4 °C plus 2 days at ambient temperature, the firmness value decreased about 80% for control fruits, and 44.3% and 54.7% for coated ones (with C-OO-TPP and A-OO-Ca coatings, respectively), concerning the initial force values (Figure 5b). 

The positive effect of coatings in maintaining figs firmness and reduce softening is in line with the results obtained for weight loss (Figure 4) and respiration rate (Figure 5). While the retention of water in the fruits results in the preservation of cell integrity [45], a lower respiration rate was related to a lower depletion of carbohydrates storage reserves in fruit tissues [48]. On the other hand, retention of firmness in coated fruits could also be explained by delayed degradation of cell wall components, especially water insoluble and NaOH insoluble pectins, due to the effect of the internal fruit atmosphere with high CO_2_ and low O_2_ content, on decreasing the activity of the cell wall hydrolases responsible for fruit softening [49]. Furthermore, calcium plays an important role in cell integrity. Therefore, the application of Ca^2+^ as crosslinking agent by spraying over the fruits covered with alginate-based emulsion could promote also the formation of calcium pectate in figs [50,51,52,53], which may explain the higher firmness values compared to the fruits coated with crosslinked chitosan-based emulsion over the storage period (Figure 5b).

Color is one of the most important attributes of fruit quality. Figure 5c,d show the surface color changes of coated and uncoated fig fruits during storage at refrigerated conditions (4 °C), and after leaving the refrigerated chamber and maintained at room temperature (25 °C) for 2 days. All samples showed a high ΔE value (>6) at 1 day of cold storage, increasing progressively until the end of that storage period, up to around 7.3 to 11.2, with non-significant differences between coated and uncoated fruits (Figure 5c). 

Along with water loss and respiration rate, color changes in fig fruits were greatly influenced by storage temperature. Thus, it was expected to have more accentuated color changes over time in fruits stored at higher temperatures. This was observed for fruits after leaving the refrigerated chamber and maintained at room temperature (25 °C) for 2 days. A substantial increase of ΔE occurred between the first and the second day at room temperature, especially for coated fruits. In fact, ΔE values of coated figs were significantly higher (*p* < 0.05) than those of control fruits (Figure 5d). The coatings induced more dark-colored pigments on the fruits surface throughout the storage period (Figure 2b). These results suggest that the chitosan or alginate-olive oil emulsions used as coatings are prone to increase the change of the external figs surface color during storage at ambient temperature, mainly when previously stored at 4 °C for at least 7 days. Indeed, some factors could influence the development of dark-colored pigments, which include changes in ascorbic acid content, sugar profiles, peroxidase activity and phenolic compounds content [54]. These factors may have been increased when coatings were applied. By the contrary, Reyes-Avalos et al. [3] results show benefits on using a bilayer alginate-chitosan emulsion film has a coating to retaining the external color of fresh figs during storage at low temperature. However, the color of the figs used by these authors is naturally dark, while the figs used in the present work present a green color, in which changes are more perceptible. 

#### 3.2.5. Total Soluble Solids and Titratable Acidity

**Figure 6:**
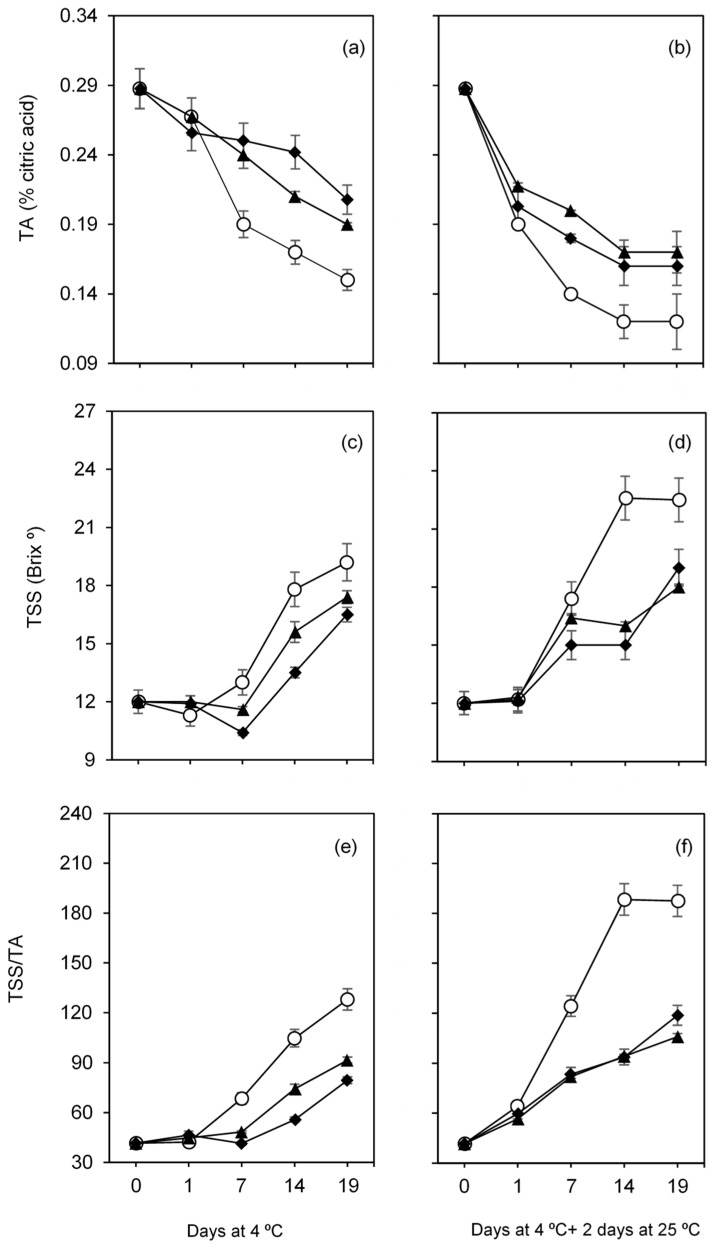
Titratable acidity (TA), total soluble solids (TSS) and maturity index (TSS/TA) of fresh figs (*Ficus carica* L. var. ´Pingo de mel´) coated (◆ C-OO-TPP and ⯅ A-OO-Ca) and uncoated (○ Ctr), under refrigeration for over 19 days at 4 °C (**a**,**c**,**e**) and after being transferred to room temperature for 2 days (**b**,**d**,**f**). Each symbol represents each experimental condition, which is associated to a line.

Coatings were effective in delaying the loss of acidity (TA, % citric acid) when comparing to uncoated fruits, both during storage under refrigeration (4 °C) (Figure 6a), and after leaving the refrigerated chamber and maintained at room temperature (25 °C) for 2 days (Figure 6b). TA values of control fruits decreased from 0.29% at day 0 to 0.12% after 19 days at 4 °C. In addition, after 19 days at 4 °C followed by 2 days at 25 °C, TA values of coated fruits were significantly higher (0.16% and 0.17%, with C-OO-TPP and A-OO-Ca coatings, respectively) than that of uncoated ones (0.12%). The effect of the coatings on acidity retention could be a result of the lower respiration rate observed in coated fruits, since organic acids are substrates for many reactions during aerobic respiration in plant cells [45].

The application of the emulsion-based coatings decreased substantially the total soluble solids (TSS, °Brix) over time when compared to uncoated fruits. TSS with a value of 12 °Brix at day 0, increased during the cold storage period for all fig groups, reaching maximum values of 19.2 °Brix, 17.4 °Brix and 16.5 °Brix for control, A-OO-Ca and C-OO-TPP fruits, respectively (Figure 6c). Interestingly, when coated figs were transferred to room temperature after the refrigerated storage period, their TSS did not increase substantially during the 2 days at 25 °C. By the contrary, for uncoated fruits, TSS increased substantially, around 88% after 14 days at 4 °C plus 2 days at 25 °C, which was maintained until 19 days at 4 °C plus 2 days at 25 °C. TSS results may be explained by the observed lower weight loss for coated fruits (Figure 4), along with the lower respiration rate, imparting a decreased maturation rate detected for coated fruits mainly during storage at room temperature. Additionally, the delayed TSS production observed on both coated figs groups (Figure 6c,d) was reflected on a much lower TSS/TA ratio (fruit maturity index), which increased significantly in the control fruits, associated with over-ripening and senescence processes (Figure 6f). The TSS/TA balance is responsible for the fruit’s flavor and is one of the main indicators of their quality [55]. Both storage conditions and coating types tested in this work were found to have a significant beneficial effect (*p* < 0.05) on fruits TSS and TA. A similar TSS/TA trend over 9 days of cold storage at 4 °C was also observed when applying chitosan coatings on another cultivar of fresh figs (“Troiano”) [56].

## 4. Conclusions

The present study was focused on the development and application of crosslinked edible coatings from chitosan (C-OO-TPP) and alginate (A-OO-Ca) based emulsions with olive oil, in order to improve the quality and shelf life of fresh figs (*F. carica* var. ´Pingo de mel´). Both emulsions exhibited a good stability at room temperature (25 °C), however, it is suggested a pre-storage at 4 °C to increase its viscosity that ultimately would favor the adhesion on the fruit’s surface as a coating. 

The application of both crosslinked chitosan-based and alginate-based emulsions as coatings was beneficial in decreasing water loss, respiration rate, firmness, fungal disease incidence and fruit maturity index of figs, when comparing to uncoated ones. From the results obtained, it is envisaged a fruits shelf life between 14 and 19 days under refrigeration at 4 °C that may be followed up to 2 days at ambient temperature, higher than that foresee for uncoated fruits (less than 14 days at 4 °C followed up to 2 days at ambient temperature). In using the coatings for avoiding fungal decay of fresh figs, it may be convenient a storage for a shorter period (14 days) or short-distance transport and distribution. However, for longer storage periods, the use of coatings functionalized with antioxidants to control color change is advised. In this case, less expensive edible oils than olive oil should also be tested. 

## Figures and Tables

**Table 1:** Parameters of modified Cross model (Equation (1)) for the studied emulsions at 4° and 25 °C temperatures. Values are expressed as mean (±standard deviation). C-OO = water-in-oil emulsion (chitosan and olive oil), A-OO = water-in-oil emulsion (chitosan and olive oil). η0 = zero-shear of viscosity, λ = relaxation time, m = 1 − n (n is the flow index), *R*^2^: Coefficient of determination, SDE: Standard deviation of the estimate, MRE: Mean relative error.

Sample	T (°C)	Cross Model Parameters			*R* ^2^	SDE	MRE
		Ƞ0 (Pa·s)	λ (s)	m			
C-OO	4	0.612 (0.003)	0.003 (0.0001)	0.711 (0.017)	0.997	0.025	3.2
	25	0.283 (0.004)	0.001 (0.0001)	0.609 (0.065)	0.971	0.024	4.7
A-OO	4	0.620 (0.002)	0.010 (0.0002)	0.717 (0.009)	0.999	0.023	3.1
	25	0.346 (0.004)	0.004 (0.0003)	0.781 (0.045)	0.990	0.032	6.1

## Data Availability

Not applicable.
